# Pilot Assessment of RNA Stabilization Methods for Influenza A Virus in Swine Oral Fluids

**DOI:** 10.3390/pathogens15040439

**Published:** 2026-04-18

**Authors:** Berenice Munguía-Ramírez, Betsy Armenta-Leyva, Luis Giménez-Lirola, Yanqi Zhang, Bailey Arruda, Giovana Ciacci-Zanella, Jeffrey Zimmerman

**Affiliations:** 1 Department of Veterinary Diagnostic and Production Animal Medicine, College of Veterinary Medicine, Iowa State University, 1800 Christensen Drive, Ames, IA 50011-1134, USA; betsyarl@iastate.edu (B.A.-L.); luisggl@iastate.edu (L.G.-L.); jjzimm@iastate.edu (J.Z.); 2 Department of Statistics, College of Liberal Arts and Sciences, Iowa State University, 2438 Osborn Drive, Ames, IA 50011-4009, USA; zyq1998@iastate.edu; 3 Virus and Prion Research Unit, National Animal Disease Center, Agricultural Research Service, United States Department of Agriculture, Ames, IA 50010-9602, USA; bailey.arruda@usda.gov (B.A.); giovana.zanella@usda.gov (G.C.-Z.); 4 Department of Veterinary Microbiology and Preventive Medicine, College of Veterinary Medicine, Iowa State University, 1800 Christensen Drive, Ames, IA 50011-1134, USA

**Keywords:** swine influenza, oral fluids, viral RNA, stabilizers, carbohydrates

## Abstract

Influenza A virus (IAV) surveillance in swine relies heavily on molecular detection, yet RNA stability in diagnostic specimens such as oral fluids can be rapidly compromised when cold-chain conditions are not maintained. This pilot study evaluated the ability of four molecular-grade carbohydrates (20% trehalose, sorbitol, sucrose, and mannitol) and two commercial nucleic acid stabilizers (PrimeStore^®^ MTM and RNA*later*^®^) to preserve RT-qPCR-detectable IAV RNA in swine oral fluids exposed to field-relevant stress conditions. Oral fluid samples collected from pigs experimentally infected with H1N2 (Study 1: *n* = 150; DPIs 2, 3, 4) or with H1N2 and H3N2 (Study 2: *n* = 58; DPI 5) were subjected to storage at 25 °C for up to 144 h (Study 1) or 2, 5, 10, or 15 freeze–thaw cycles (Study 2), with DPIs (Study 1) or subtypes (Study 2) serving as biological replicates, given the limited sample size. IAV detection was quantified as efficiency standardized Cq values (ECq) and analyzed using a linear mixed-effects model. Overall, both carbohydrates (trehalose, sorbitol, sucrose) and commercial stabilizers maintained higher ECq values than untreated oral fluids under both thermal and freeze–thaw stress conditions. Due to the limited sample size, these findings should be interpreted cautiously, yet they demonstrate the potential utility of carbohydrates as a low-cost, non-inactivating alternative for stabilizing IAV RNA in field-collected oral fluids.

## 1. Introduction

Influenza A virus (IAV) is an enveloped virus with a single-stranded, negative-sense RNA genome organized in eight segments [1]. IAV subtyping is based on hemagglutinin (H1-18) and neuraminidase (N1-11) surface glycoproteins, with H1N1, H1N2, and H3N2 currently the most common subtypes in swine populations [2]. IAV has a significant impact on swine health worldwide, imposing losses estimated at $3.23 to $10.31 USD per pig [3]. Surveillance of swine populations plays an important role in guiding IAV prevention and control measures [4], and both experimental and field studies have shown that oral fluids are a practical and effective surveillance specimen for IAV RNA detection [5,6]. Indeed, pen-based oral fluids often provide higher detection rates than individual pig nasal swabs [7,8].

In the laboratory, molecular testing is dependent on the quality of the nucleic acid in the sample. IAV is of particular concern in this regard because RNA is vulnerable to both base-catalyzed hydrolysis and to the ribonucleases ubiquitous in the environment [9]. The need to maintain viral integrity in field specimens was recognized as early as 1929, and protective strategies included freeze-drying [10] and the use of viral transport media [11]. In the 1960s, carbohydrates were found to be effective virus stabilizers [12,13,14,15,16]. Thus, Gupta et al. (1996) [17] reported that 25% sucrose, 10% trehalose, or 10% sorbitol maintained respiratory syncytial virus infectivity at ambient temperatures and through multiple freeze–thaw cycles. Similarly, Kissmann et al. (2008) [18] found that 10% mannitol, 20% trehalose, 20% sorbitol, or 20% sucrose (20%) preserved the infectivity of recombinant measles vaccine virus—even under thermal stress (55 °C) and mildly acidic conditions (pH 5.5). As molecular testing developed, commercial products based on salts, chaotropic agents, or other components were added to diagnostic samples to inhibit nucleases and maintain nucleic acid integrity from the field to the laboratory. Thus, Flinders Technology Associates (FTA)^®^ cards were introduced in the 1980s and, in the latter 1990s, aqueous storage matrices, e.g., RNA*later*^®^ (a stabilization solution designed to preserve RNA without inactivating infectious agents), PrimeStore^®^ MTM (a molecular transport medium designed to inactivate infectious pathogens and stabilize nucleic acids), and DNA/RNA Shield^TM^ (a stabilization solution for nucleic acids in biological samples).

Carbohydrates, despite their low cost, ready availability, and reported efficacy, have not been fully investigated for their potential to preserve viral nucleic acid in diagnostic specimens. Thus, the objective of this study was to compare molecular biology-grade carbohydrates (trehalose, sorbitol, sucrose, and mannitol) and two commercial storage products: PrimeStore^®^ MTM (Longhorn Vaccines and Diagnostics, Bethesda, MD, USA) and RNA*later*^®^ (Thermo Fisher Scientific, Waltham, MA, USA), in terms of their capacity to preserve RT-qPCR detectable IAV RNA in swine oral fluid samples exposed to less-than-ideal storage conditions.

## 2. Materials and Methods

### 2.1. Experimental Design

Two studies were performed to evaluate the detection of IAV RNA in oral fluid samples treated with molecular-grade carbohydrates (20% *w*/*v*), i.e., (1) trehalose, (2) sorbitol, (3) sucrose, (4) mannitol, and commercial nucleic acid stabilizers: (5) PrimeStore^®^ MTM (Longhorn Vaccines and Diagnostics, Bethesda, MD, USA), and (6) RNA*later*^®^ (Thermo Fisher Scientific, Waltham, MA, USA). In Study 1 (temperature × time), IAV detection was evaluated in treated oral fluid samples held at 25 °C for up to 144 h. In Study 2 (freeze–thaw cycles), IAV detection was evaluated in treated oral fluid samples subjected to freeze–thaw cycles (2, 5, 10, 15). In both studies, RT-qPCR test results were normalized as efficiency standardized Cqs (ECqs), and IAV RNA stability was evaluated using a linear mixed-effects regression model with the lmer() function from the lme4 package in R v4.5.1 [19,20].

### 2.2. Influenza A Viruses

The two virus isolates used in this study were collected through the USDA Influenza A Virus in Swine Surveillance Program [4], i.e., A/swine/Ohio/A02750994/2022 (H1N2) (GenBank OP903621.1) or A/swine/Iowa/A02636454/2022 (H3N2) (GenBank OM935893.1). To prepare the stock solutions, the virus was propagated as described by Gauger and Zhang (2020) [21]. Briefly, Madin-Darby canine kidney (MDCK) cells were seeded into 500 cm^2^ triple flasks (Nunc™ TripleFlask™, Thermo Fisher Scientific, Waltham, MA, USA). When cells reached 80–90% confluence, cells were inoculated with viral isolates at a multiplicity of infection of 0.01 and incubated for 1 h at 37 °C to allow viral adsorption. Thereafter, the inoculum was removed, and cells were washed twice with phosphate-buffered saline (PBS). Cells were incubated in Opti-MEM™ medium (Life Technologies, Burlington, ON, Canada) at 37 °C with 5% CO_2_ for 2 days and monitored daily for cytopathic effect (CPE). When ~80% of the cells exhibited CPE, flasks were subjected to two freeze–thaw cycles (−80 °C), after which culture supernatants were harvested and clarified by centrifugation (1800× *g* for 30 min).

Endpoint titration of the working virus stocks was determined in confluent MDCK cells grown in 96-well plates, as described by Gauger and Vincent (2014) [22]. In brief, the virus inoculum was 10-fold serially diluted in triplicate using Opti-MEM™ medium (Life Technologies, Burlington, ON, Canada) supplemented with antibiotics/antimycotics and 1 µg/mL tosyl sulfonyl phenylalanyl chloromethyl ketone (TPCK)-treated trypsin (Worthington Biochemical Corp., Lakewood, NJ, USA) to facilitate viral entry and replication in cells [23]. The endpoint was determined as the highest dilution showing CPE. The virus titer was calculated as the median 50% tissue culture infectious dose (TCID_50_) per mL using the Reed and Muench (1938) method.

### 2.3. IAV Animal Study

The pigs used in this study were farrowed in research facilities on the USDA National Centers for Animal Health campus (Ames, IA, USA) and cared for in compliance with the guidelines established by the Institutional Animal Care and Use Committee of the USDA-ARS National Animal Disease Center (NADC). At three weeks of age, pigs (*n* = 16) were tagged and placed under biosafety level 2 conditions in two separate rooms: one for the group to be inoculated with H1N2 (*n* = 8 pigs) and one for the H3N2 group (*n* = 8 pigs). Within each room, pigs were housed in two pens holding four pigs each. At the termination of the study (DPI 21), pigs were humanely euthanized with a lethal intravenous dose of pentobarbital (Fatal Plus; Vortech Pharmaceuticals, Dearborn, MI, USA).

On DPI 0, each pig was administered 2 mL (1 mL in each naris) of virus inoculum containing 5.6 × 10^4^ (H1N2) or 1.78 × 10^5^ (H3N2) TCID_50_ per mL using an intranasal atomization device (MAD Nasal^TM^, Teleflex, NC, USA). On day post-inoculation (DPI) −5 and 21, pigs were bled, and serum was tested for antibodies against the IAV nucleoprotein (Swine Influenza Virus Antibody Test, IDEXX, Westbrook, ME, USA). On DPI 0, 3, 5, and 7, nasal swab samples were collected (FLOQSwabs^®^, Copan, Murrieta, CA, USA), after which swabs were placed in a tube with 2 mL of minimum-essential-medium, and later tested for IAV RNA (MagMax Viral RNA isolation kit and VetMAX™ Gold SIV Detection Kit, ThermoFisher Scientific, Waltham, MA, USA) at NADC. On DPIs −4, −3, −2, and −1, pigs were trained for oral fluid collection by suspending cotton ropes (1.3 cm 3-strand twisted 100% cotton rope, Skydog Rigging Equipment, Lake in the Hills, IL, USA) in the middle of the pens from a chain (~30 min). On DPIs 0 to 7, oral fluid samples were collected from each pen (*n* = 2 per room), packaged as per federal regulations (49 CFR § 173.199s), and transported to the Iowa State University Veterinary Diagnostic Laboratory (ISU-VDL, Ames, IA, USA). Upon arrival at the ISU-VDL, oral fluids were pooled by room (H1N2 or H3N2) by combining the two samples collected per room on each DPI (~50 mL per sample) to obtain sufficient total volume for Studies 1 and 2. Samples from DPIs 0, 1, 6, and 7 were divided into 1 mL aliquots (Falcon^®^ 5 mL round-bottom polystyrene test tube, Corning^®^, Corning, NY, USA) and stored at −80 °C. Samples from DPIs 2, 3, 4, and 5 were processed for Studies 1 and 2 as described in the following sections.

### 2.4. IAV RNA Stabilizer Treatments

Study 1 (temperature × time) and Study 2 (freeze–thaw cycles) compared 6 prospective stabilizers in terms of their effect on the detection of IAV RNA, including 4 molecular-grade carbohydrates, i.e., trehalose (D-(+)-Trehalose dihydrate; Sigma-Aldrich^®^, Saint Louis, MO, USA), sorbitol (D-Sorbitol; MP Biomedicals^TM^, LLC, Solon, OH, USA), sucrose (D(+)-Saccharose; Sigma-Aldrich^®^, Saint Louis, MO, USA), mannitol (D-Mannitol; Sigma-Aldrich^®^, Saint Louis, MO, USA), and 2 commercial products, i.e., PrimeStore^®^ MTM (Longhorn Vaccines and Diagnostics, Bethesda, MD, USA) and RNA*later*^®^ (Thermo Fisher Scientific, Waltham, MA, USA). Prior to receipt of the oral fluid samples at the ISU-VDL, 5 mL tubes (Falcon^®^ round-bottom polystyrene test tubes, Corning^®^, Corning, NY, USA) were labeled and loaded with stabilizer. Tubes containing molecular-grade carbohydrates were prepared to achieve a final stabilizer concentration of 20% *w*/*v* (0.2 g/mL of oral fluid). Tubes with commercial products were prepared according to the manufacturer’s instructions, i.e., 3 mL of PrimeStore^®^ MTM and 2.6 mL of RNA*later*^®^ per 1 mL of oral fluid.

### 2.5. Evaluation of Stabilizers

#### 2.5.1. Study 1: (Temperature × Time)

Study 1 evaluated IAV RNA detection in oral fluid samples subjected to one of seven temperature × time conditions, i.e., 25 °C for 12 h, 24 h, 48 h, 72 h, 96 h, 120 h, 144 h. Because the available oral fluid volume was insufficient to generate 1 mL aliquots for all treatments and experimental conditions, Study 1 was limited to oral fluid samples from the H1N2 group collected on DPIs 2, 3, and 4, with DPIs treated as biological replicates in the analysis. Upon arrival at the laboratory on each DPI, oral fluids were pooled by room, vortexed, and added (1 mL) to the tubes containing stabilizers. Thereafter, tubes were vortexed for 10 s to ensure homogenization and stored at −80 °C. Controls (*n* = 8) for each DPI included untreated controls, i.e., oral fluid aliquots without stabilizer for each temperature × time condition, plus one reference oral fluid sample without stabilizer that remained stored at −80 °C. Thus, Study 1 was based on 150 samples, i.e., [(6 treated oral fluids + 1 untreated control oral fluid) × (7 time points) + (1 reference control oral fluid held at −80 °C)] × (3 DPIs).

**Figure 1:**
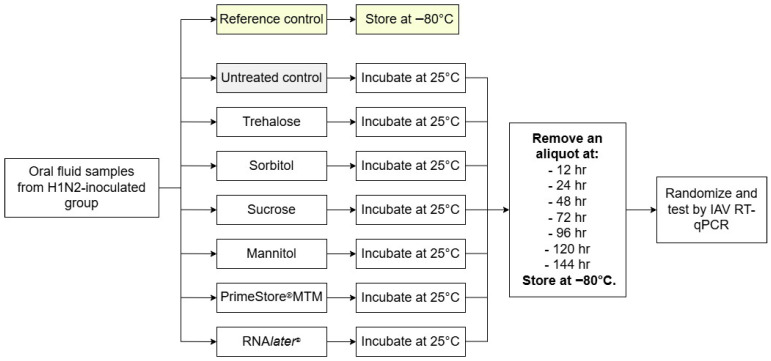
Experimental design for Study 1 (temperature × time). On each DPI (2, 3, 4), oral fluid samples collected from H1N2-infected pigs were treated with one of six stabilizers (trehalose, sorbitol, sucrose, mannitol, PrimeStore^®^ MTM, or RNA*later*^®^) and held at 25 °C. Samples were removed from the incubator at 12 h, 24 h, 48 h, 72 h, 96 h, 120 h, and 144 h (*n* = 21 per time point) and then stored at −80 °C until tested.

In Study 1, all treated samples and treatment controls (*n* = 147) were held overnight at 4 °C to thaw. As shown in Figure 1, study 1 was initiated (time zero) when samples were placed in a 25 °C incubator (NAPCO 6301-0 Precision Scientific CO_2_ Incubator, Thermo Fisher Scientific, Waltham, MA, USA) monitored using a traceable digital thermometer (Fisherbrand, Fisher Scientific, Pittsburgh, PA, USA). The recorded temperature ranged from 25.5 °C to 26.1 °C over the course of the experiment.

Thereafter, samples (*n* = 21 per time point) were removed from the incubator at 12 h, 24 h, 48 h, 72 h, 96 h, 120 h, and 144 h and then stored at −80 °C. For testing, all samples (*n* = 150) were thawed at 4 °C overnight and then tested for IAV RNA by RT-qPCR (Section 2.6).

#### 2.5.2. Study 2: Freeze–Thaw

Study 2 evaluated IAV RNA detection in oral fluid samples subjected to 2, 5, 10, or 15 freeze–thaw cycles. Oral fluids collected on DPI 5 from the H1N2 and H3N2 groups were used, each providing sufficient volume to generate 1 mL aliquots for all treatments and experimental conditions. IAV subtypes H1N2 and H3N2 were treated as biological replicates in the analysis. Upon arrival at the laboratory, oral fluids collected on DPI 5 from H1N2 and H3N2 groups were pooled within group, vortexed, and added (1 mL) to tubes randomly assigned to stabilizer treatment. Thereafter, tubes were vortexed for 10 s to ensure homogenization and then stored at −80 °C. Controls for each subtype (*n* = 5) included one untreated control, i.e., oral fluid aliquots without stabilizer for each freeze–thaw condition, plus one reference oral fluid sample without stabilizer that remained stored at −80 °C. Thus, Study 2 comprised a total of 58 samples, i.e., [(6 treated oral fluids + 1 untreated control oral fluid) × (4 freeze–thaw conditions) + (1 reference control oral fluid held at −80 °C)] × (2 subtype groups).

**Figure 2:**
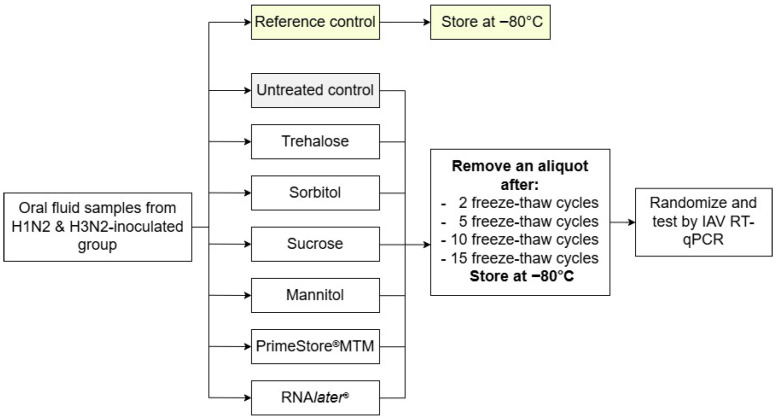
Experimental design for Study 2 (freeze–thaw cycles). Oral fluid samples collected on DPI 5 from H1N2- and H3N2-infected groups were treated with one of six stabilizers (trehalose, sorbitol, sucrose, mannitol, PrimeStore^®^ MTM, or RNA*later*^®^), subjected to 2, 5, 10, or 15 freeze–thaw cycles, and then stored at −80 °C to complete the last cycle.

As shown in Figure 2, to perform Study 2, all treated samples and treatment controls (*n* = 56) were subjected to their respective freeze–thaw cycles (2, 5, 10, or 15) by placing them at −80 °C in the morning and then 4 °C overnight. After completing the penultimate cycle, samples were stored at −80 °C. Immediately prior to testing, all samples (*n* = 58) were placed at 4 °C overnight and then tested for IAV RNA by RT-qPCR (Section 2.6).

### 2.6. Oral Fluid IAV RT-qPCR Testing

All laboratory procedures were conducted within a certified biosafety cabinet using calibrated pipettes that were within their valid certification period. Total nucleic acids were extracted from oral fluid samples using the MagMAX™ CORE Nucleic Acid Purification Kit (Thermo Fisher Scientific, Waltham, MA, USA) following the manufacturer’s protocol with Xeno™ RNA Control (Thermo Fisher Scientific, Waltham, MA, USA) spiked into the lysis buffer. Nucleic acid extraction was performed on a KingFisher™ Flex automated purification system (Thermo Fisher Scientific, Waltham, MA, USA). Detection of IAV RNA was performed according to the manufacturer’s instructions using the VetMAX™-Gold SIV Detection Kit (Thermo Fisher Scientific, Waltham, MA, USA). Each RT-qPCR reaction (25 µL) included 2X Multiplex RT-PCR Buffer (12.5 µL), 10X Multiplex RT-PCR Enzyme Mix (2.5 µL), Influenza Virus Primer Probe Mix (1.0 µL), nuclease-free water (1.0 µL), and nucleic acid extract (8 µL). RT-qPCR reactions were carried out on an ABI 7500 Real-Time PCR System (Applied Biosystems, Foster City, CA, USA) with the following thermal cycling conditions: reverse transcription at 48 °C for 10 min, initial denaturation at 95 °C for 10 min, 40 cycles of denaturation at 95 °C for 15 s, and annealing/extension at 60 °C for 45 s. Fluorescence data were collected during the annealing/extension step of each cycle. Each RT-qPCR plate included 1 extraction positive control, 1 extraction negative control, 1 amplification positive control, 1 amplification negative control, and 4 replicates of a matrix-specific (oral fluid) IAV reference standard.

### 2.7. Statistical Analysis

For data analysis, RT-qPCR Cqs were normalized as ECqs (Equation (1)) using matrix-specific reference standards to account for plate-specific RT-qPCR efficiency [19,24].

(1) Efficiency standardized Cq (ECq) = E^−ΔCq^ = E^−(sample Cq–mean reference standard Cq)^

In Equation (1), E is the mean amplification efficiency of the 4 reference standards expressed as a ratio (a doubling at each cycle would represent an efficiency of 2), and ΔCq is the difference between the sample Cq and the mean Cq of the 4 matrix-specific reference standards included in each plate.

Analysis of ECq data for Studies 1 and 2 was conducted using a linear mixed-effects regression model with IAV ECq as the response variable using the lmer() function in the lme4 package in R v4.5.1 [20]. In Study 1, fixed effects were temperature by time, stabilizer, and their interactions; DPI was included as a random effect to account for variation across sampling days. In Study 2, fixed effects included freeze–thaw cycles, stabilizer, and their interactions; IAV subtype was included as a random effect. Because each study included −80 °C reference control aliquots derived from study-specific DPIs, intercept estimates were study-dependent and intended for within-study interpretation. Model fitting was performed using restricted maximum likelihood, and the significance of fixed effects was evaluated using Type III ANOVA. Post hoc pairwise comparisons of the least square means for each stabilizer treatment relative to the untreated oral fluids exposed to the same experimental conditions, at each storage time point (Study 1) and each freeze–thaw cycle (Study 2), with Bonferroni adjustment to control for multiple pairwise tests conducted.

## 3. Results

**Figure 3:**
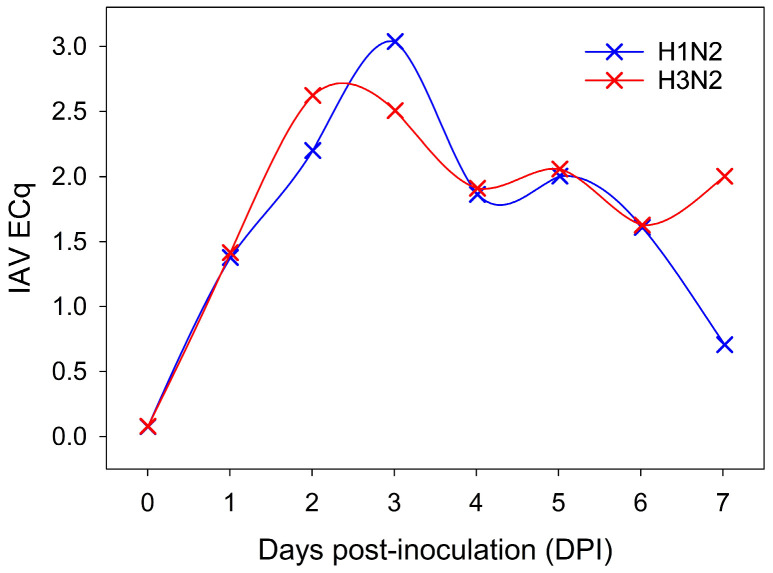
Influenza A virus (IAV) RT-qPCR results for oral fluids collected from pigs inoculated with H1N2 (blue) and H3N2 (red) on days post-inoculation (DPI) 0 to 7, reported as efficiency standardized Cqs (ECq). On each DPI, oral fluids were pooled by pen (four pigs each) and by room/group (H1N2 or H3N2).

As shown in Figure 3, IAV RNA was detected in oral fluid samples from DPIs 1 to 7 for both the H1N2 and H3N2 groups.

Compatibility of carbohydrate stabilizers with oral fluids: All carbohydrate and commercial stabilizers were easily incorporated into the oral fluid matrix with standard vortexing. Thereafter, all samples were readily pipettable except for mannitol-treated oral fluids, which gradually developed a fine sediment that interfered with pipetting. This precipitation imposed practical handling limitations and reflected formulation-specific constraints that may reduce the suitability of this carbohydrate for use in liquid oral fluid matrices.

### 3.1. Results: Study 1 (Temperature × Time Treatment)

**Figure 4:**
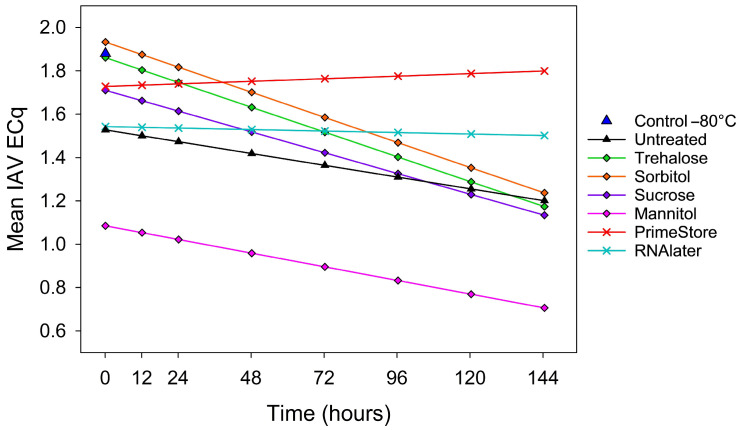
Linear mixed-effects model showing the effect of temperature (25 °C), storage time, and stabilizer treatment on the mean IAV RNA (ECq) response in oral fluids collected from pigs inoculated with H1N2. Type III ANOVA showed significant main effects of stabilizer treatment and storage time. Bonferroni-adjusted pairwise comparisons indicated that these differences were driven by mannitol, which differed significantly from the reference control held at −80 °C, PrimeStore^®^ MTM, sorbitol, sucrose, and trehalose (*p* < 0.05).

Overall, the analysis (Type III ANOVA) showed that stabilizer treatment (*p* < 0.05), storage temperature × time (*p* < 0.05), and their interactions (*p* < 0.05) affected IAV ECq values in oral fluids, with PrimeStore^®^ MTM providing the slowest decrease in IAV ECqs over time (+0.002 ECqs/h; *p* < 0.05). Compared to the reference control oral fluid held at −80 °C (y = 1.88 ECq), intercepts estimated from the linear mixed model were significantly lower (Figure 4) for mannitol (−0.79 ECq; *p* < 0.05), RNA*later^®^* (−0.34 ECq; *p* = 0.01), and the untreated oral fluid (−0.35 ECq; *p* < 0.05). Compared to the untreated control oral fluid, pairwise comparisons using Bonferroni-adjusted contrasts of the IAV ECq least square means for each time point showed that no stabilizer differed significantly from the untreated oral fluids across all time points, i.e., 12 to 144 h (*p* > 0.05). However, as a general trend over time, mannitol consistently produced lower IAV ECqs than the untreated oral fluid, whereas trehalose, sorbitol, sucrose, PrimeStore^®^ MTM, and RNA*later*^®^ tended to yield higher IAV ECqs (Figure 4).

### 3.2. Results: Study 2 (Freeze–Thaw Treatment)

Both H1N2 and H3N2 IAV subtypes showed similar ECq responses across stabilizers and freeze–thaw cycles. The analysis (Type III ANOVA) revealed that the number of freeze–thaw cycles (*p* < 0.05), the stabilizer treatment (*p* < 0.05), and their interaction (*p* < 0.05) had a significant effect on IAV ECqs. However, further analysis (linear mixed model) showed that the only stabilizer treatment with a significant interaction with freeze–thaw cycles was RNA*later*^®^ (*p* < 0.05). That is, the estimated ECq in RNA*later*^®^-treated samples increased by 0.041 ECqs per cycle.

**Figure 5:**
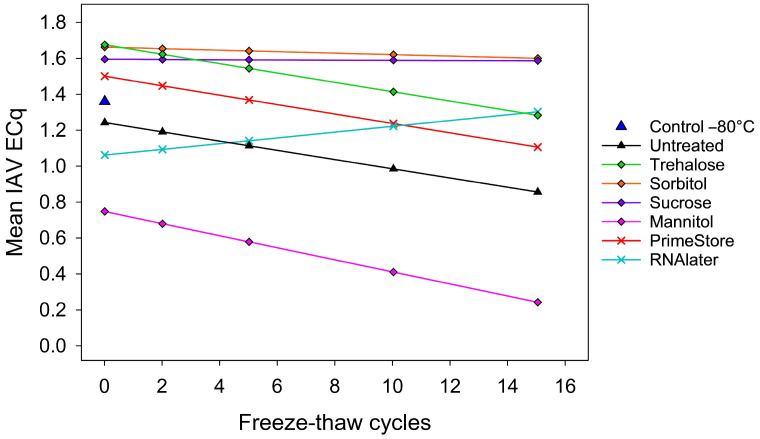
Linear mixed-effects model showing the effect of freeze–thaw cycles and stabilizer treatment on the mean IAV RNA (ECq) response in oral fluids collected at 5 DPI from pigs inoculated with H1N2 or H3N2. Treated oral fluids were assigned to one of four freeze–thaw treatments: 2, 5, 10, and 15 cycles. A freeze–thaw cycle consisted of storing samples at −80 °C and then thawing them at 4 °C overnight. Type III ANOVA showed significant main effects of stabilizer treatment and freeze–thaw cycles. Bonferroni-adjusted pairwise comparisons indicated that these differences were primarily driven by mannitol, which differed significantly from the reference control held at −80 °C, PrimeStore^®^ MTM, sorbitol, sucrose, and trehalose (*p* < 0.05). In addition, RNA*later^®^* differed significantly from sorbitol and sucrose (*p* < 0.05).

As shown in Figure 5, compared to the reference control oral fluid held at −80 °C (y = 1.36 ECq), the intercept estimated from the linear mixed model was significantly lower for mannitol (−0.61 ECq; *p* < 0.05). As a general trend, trehalose, sorbitol, sucrose, PrimeStore^®^ MTM, and RNA*later*^®^ yielded higher IAV ECq values than the untreated oral fluid. However, compared to the untreated control oral fluid, pairwise comparisons using Bonferroni-adjusted contrasts of the IAV ECq least square means for each freeze–thaw cycle showed that only mannitol differed significantly, i.e., consistently produced lower ECqs (*p* < 0.05), from the untreated oral fluids across freeze–thaw cycles. No other stabilizer differed significantly from the untreated oral fluid at any specific freeze–thaw cycle.

## 4. Discussion

Physicochemical stressors, including changes in pH, shear/mechanical stresses, freeze–thaw cycles, and oxidative denaturation [25], cause unfolding of viral epitopes, loss of capsid integrity, and major conformational rearrangements that lead to virus degradation [26]. Simply put, when viral capsid integrity is compromised, RNases in the environment or sample matrix are able to access and degrade viral RNA [27]. Many of these stressors can be understood in terms of pressure- and temperature-dependent shifts in the Gibbs free energy associated with viral protein folding [25,28]. Because the energy difference between the folded (active) and unfolded (inactive) states is small (~5 to 20 kcal/mol) [29,30], even slight increases in temperature can shift the equilibrium toward the unfolded state, leading to protein denaturation and viral aggregation.

At subzero temperatures (<0 °C), additional stresses arise from temperature fluctuations relative to the glass transition temperature (Tg) of the freeze-concentrated phase. The glass transition temperature is the point at which the frozen matrix shifts from a rigid, amorphous “glassy” state with severely restricted molecular mobility to a more dynamic, “rubbery” state that permits diffusion-driven chemical and biochemical reactions [31]. Viral particles stored at temperatures above Tg are exposed to environments characterized by solute crowding, pH shifts, and increased molecular mobility, all of which accelerate protein and nucleic acid degradation [32,33]. Importantly, Tg is not a fixed temperature, but varies with matrix composition [34]; for which reason the effective Tg of complex matrices, e.g., swine oral fluids, is poorly defined. Consequently, storage at ultra-low temperatures (−70 to −80 °C) is performed to ensure samples remain below Tg, hence minimizing molecular mobility, residual enzymatic activity, and RNA degradation during long-term storage [35,36].

In commercial vaccine formulation, mitigation of stress-induced viral protein destabilization commonly relies on the inclusion of excipients such as disaccharides (e.g., sucrose, trehalose), polyols (e.g., sorbitol, mannitol), and cryoprotectants (e.g., glycerol, alginate) [25,33]. Basically, excipients preserve viral particle integrity by increasing Tg, reducing molecular mobility, and other physicochemical interactions [37]. Non-ionic disaccharides, e.g., sucrose and trehalose, do not directly modify the intrinsic Gibbs free energy of viral proteins. Instead, they stabilize the native (folded) state by increasing the energy barrier to unfolding, thereby indirectly reducing the probability of denaturation and aggregation [38]. In addition, non-ionic disaccharides elevate the Tg by forming amorphous, vitrified matrices during freezing that immobilize water and solutes, suppress buffer crystallization, and limit pH excursions [39]. Thus, sucrose and trehalose are able to protect viral proteins during freeze–thaw cycles and temperature fluctuations by restricting conformational flexibility and intermolecular interactions that promote unfolding and aggregation [16,17,18,40]. Beyond disaccharides, some sugar alcohols (polyols), such as sorbitol and mannitol, can mitigate stress-induced viral instability [18], but their effectiveness varies by formulation, matrix, and storage conditions [25]. Several reports suggested that mannitol performed inconsistently in liquid systems, particularly during cooling or storage, where crystallization potentially affects virus stability [16].

Oral fluid is a valuable specimen for IAV surveillance due to its ease of collection and ability to represent group-level infection status. However, it represents a complex biological matrix in which viral RNA stability is rapidly compromised when samples are not properly maintained [41,42]. The pilot study presented herein was designed to evaluate stabilizers commonly used in vaccine formulations for their ability to preserve IAV RNA in swine oral fluids under two field-relevant stress conditions: prolonged exposure to 25 °C and repeated freeze–thaw cycles. These conditions reflect realistic, i.e., less than perfect, field sample handling scenarios. Environmental humidity was not controlled or measured and was considered a variable likely to fluctuate during real-world field transport and handling. Future studies could further assess the influence of controlled humidity on stabilizer performance and viral RNA stability.

For the analysis, raw RT-qPCR results were normalized as ECqs to account for plate-specific amplification efficiency. As described elsewhere [19,24], normalization compensates for inherent variation in the testing process by expressing the result in the context of a common standard, thereby improving the repeatability of the assay. Normalization is widely used in research studies [24] but can likewise be used in diagnostic testing [19].

Under both static (25 °C) and freeze–thaw conditions, untreated samples exhibited a progressive reduction in IAV ECq values over time. Oral fluids treated with trehalose, sorbitol, and sucrose and held at 25 °C likewise exhibited time-dependent degradation but consistently yielded higher IAV ECq values than untreated oral fluids. In contrast, mannitol did not provide measurable stabilization under these conditions. Regarding commercial stabilizers, both RNA*later*^®^ and PrimeStore^®^ MTM maintained higher IAV RNA detectability than the untreated oral fluids over time.

Under repeated freeze–thaw stress, trehalose, sorbitol, and sucrose demonstrated robust protective effects, maintaining higher ECq values than untreated controls. Sorbitol and sucrose, in particular, preserved relatively stable ECq values even after 15 freeze–thaw cycles, whereas mannitol showed no evidence of IAV RNA stabilization. Among commercial products, both RNA*later*^®^ and PrimeStore^®^ MTM preserved IAV RNA detectability across freeze–thaw cycles; however, the stabilizing effect of PrimeStore^®^ MTM declined as the number of cycles increased.

Overall, commercial stabilizers provided more protection against IAV RNA temperature × time degradation, and carbohydrate stabilizers provided more effective protection against freeze–thaw effects. Notably, trehalose, sorbitol, or sucrose effectively stabilized IAV RNA in oral fluid under both thermal and freeze–thaw stress conditions, demonstrating that simple carbohydrate-based formulations can preserve viral RNA in the absence of proprietary commercial agents. In addition, carbohydrates offer substantial cost advantages [40]. As of 2025, the estimated per-sample cost of the carbohydrates evaluated in this study ranged from $0.018 to $0.738 USD, compared with $5.18 to $5.37 USD per sample for the proprietary stabilizers tested. In addition, unlike most commercial stabilizers (except for RNA*later*^®^), carbohydrates do not inactivate viruses, thereby enabling downstream diagnostic analyses such as infectivity or culture-based assays. Taken together, the combination of low cost, non-inactivating properties, and stabilization of IAV RNA makes carbohydrate-based formulations a practical alternative for preserving viral RNA in field-collected swine oral fluid samples destined for veterinary diagnostic laboratories.

## 5. Conclusions

This pilot study demonstrated that trehalose, sorbitol, and sucrose effectively stabilize RT-qPCR detectable IAV RNA in swine oral fluid samples under suboptimal storage conditions, including 25 °C storage over time (≤72 h) and up to 15 freeze–thaw cycles. As for mannitol, its lower performance and handling difficulty reduce its practical utility for applied diagnostic workflows. Limitations of this study included sample size, including the fact that it was not possible to evaluate both H1N2 and H3N2 IAV subtypes under all conditions. Accordingly, these findings should be interpreted cautiously and within the context of the study’s scope. Future studies on virus preservation and diagnostic enhancement will focus on optimizing carbohydrate concentrations, investigating additional compounds commonly used in the vaccine industry to achieve enhanced viral RNA stabilization, and the inclusion of other viruses of economic importance in the swine industry.

## Data Availability

All raw ECq values obtained for both Studies 1 and 2, along with corresponding treatment groups, DPIs, and subtype information, are provided in the Supplementary Materials. Further inquiries can be directed to the corresponding author.

## Supplementary Materials

The following supporting information can be downloaded at: https://www.mdpi.com/article/10.3390/pathogens15040439/s1.
